# Identification of differentially expressed microRNAs between *Bacillus thuringiensis* Cry1Ab-resistant and -susceptible strains of *Ostrinia furnacalis*

**DOI:** 10.1038/srep15461

**Published:** 2015-10-21

**Authors:** Li-Na Xu, Ying-Hui Ling, Yue-Qin Wang, Zhen-Ying Wang, Ben-Jin Hu, Zi-Yan Zhou, Fei Hu, Kang-Lai He

**Affiliations:** 1 Institute of Plant Protection and Agro-Products Safety, Anhui Academy of Agricultural Sciences, Hefei, Anhui 230031, China; 2 The State Key Laboratory for Biology of Plant Diseases and Insect Pests, Institute of Plant Protection, Chinese Academy of Agricultural Sciences, Beijing 100193, China; 3 College of Animal Science and Technology, Anhui Agricultural University, Hefei, Anhui 230036, China

## Abstract

The Asian corn borer (ACB), *Ostrinia furnacalis* (Guenée), can develop strong resistance to Cry1Ab, the most widely commercialized Cry toxin for Bt maize worldwide. It is essential to understand the mechanism of resistance for management of this species, but information on the post-transcriptional regulation of Bt resistance in this target insect is limited. In the present study, RNA was extracted from the ACB in various larval stages (1–5 instar) from Cry1Ab-sensitive (ACB-BtS) and -resistant (ACB-AbR) strains, each of which included two biological replicates. Using Illumina sequencing, a total of 23,809,890 high-quality reads were collected from the four ACB libraries. The numbers of known microRNAs (miRNAs) were 302 and 395 for ACB-BtS and 268 and 287 for ACB-AbR. Using Mireap software, we identified 32 and 16 potential novel miRNAs for ACB-BtS and 18 and 22 for ACB-AbR. Among them, 21 known and 1 novel miRNAs had significantly different expression between ACB-BtS and ACB-AbR. Several miRNAs were observed to target potential Bt receptor genes, such as aminopeptidase N and cadherin-like protein. The glycosylphosphatidylinositol-anchor biosynthetic process and ABC transporters pathway were identified through Gene Ontology and KEGG pathway analysis of target genes of the differentially expressed miRNAs.

The Asian corn borer (ACB), *Ostrinia furnacalis* (Guenée) (Lepidoptera: Crambidae), is the most destructive corn-stalk-boring pest in Asia, particularly in China and the Philippines. Estimated yield losses from this pest are 10–20% and may exceed 30%; in some cases, entire harvests are lost in an outbreak year^1^,^2^. Field trials in China have demonstrated that Cry1Ab-expressing maize MON810 and Bt11 have the potential to effectively control the ACB and other lepidopteron pests^3^,^4^. However, resistance of the ACB to the Cry1Ab toxin has been found to increase more than 100-fold after 35 generations using artificial diets containing the Cry1Ab protein under laboratory conditions^5^. Moreover, the Cry1Ab-resistant strain of the ACB can survive on Cry1Ab-expressing maize silk after 51 generations of selection^6^. Understanding the mechanism of the ACB resistance to Cry1Ab is the key to developing resistance management strategies and delaying the resistance evolution of target insects.

It has been reported that the mutation of aminopeptidase N (APN) genes^7^, cadherin-like protein (CAD)^8^, and the different expression of APN^7^, V-type proton, ATPase catalytic subunit A, heat shock 70 kDa^9^, and alkaline phosphatase (ALP)^10^ could contribute to the development of Cry1Ab/Cry1Ac resistance of ACB. Expression of the genes is regulated at both transcriptional and post-transcriptional levels. MicroRNAs (miRNAs) are known to be a key component in post-transcriptional gene expression regulation in many species. miRNAs are endogenous non-protein-coding RNAs and negatively regulate gene expression by complementarily binding to the ORF or UTR region of target messenger RNAs. Since they were first reported in humans, fruit flies, and nematodes, these vital participants in post-transcriptional gene regulation have received increasing attention^11^. miRNAs were first identified in an insect species through studies in *Drosophila*^12^. *Drosophila* miRNAs have revealed distinct roles in not only many important developmental events in insects but also various conserved mechanisms in animals, such as ageing^13^, apoptosis^14^, cell growth and proliferation^15^, carbon dioxide receptor formation^16^, regulation of metabolism^17^, neurodegeneration^18^, and the Wnt/wingless signalling pathway^19^.

Accumulating evidence in recent years suggests that miRNAs have effects on insect-pathogen interactions, although such studies are limited compared with research on insect development. Infection with *Autographa califormica* multiple nucleo-polyhedrosis virus (AcMNPV) in *Spodoptera frugiperda* (Sf9) cells resulted in a large number of changes in miRNA expression, such as the upregulation miR-184, miR-998 and miR-10 at 24 h post-infection (hpi) and, for some of these miRNAs, downregulation at 72 hpi^20^. In *Bombyx mori* larvae infected with *B. mori* cytoplasmic polyhedrosis virus (BmCPV), 58 miRNAs were found to be significantly upregulated or downregulated in the mid-gut compared with non-infected larvae at 72 and 96 hpi^21^. Similar to viruses, bacterial infections can lead to changes in cellular miRNAs in different insect species. Comparison of the small RNA libraries of *Wolbachia*-infected and uninfected *Tetranychus urticae* showed that *Wolbachia* infection significantly regulated miRNAs in both females and males, with an overall suppression of miRNAs in *Wolbachia*-infected libraries^22^. Microarray analysis revealed that several mosquito miRNAs were differentially expressed in *Wolbachia*-infected and non-infected mosquitoes, with two miRNAs, aae-miR-2940 and -309a, showing higher abundance in infected ones^23^. Function of another *Wolbachia*-induced cellular miRNA, aae-miR-12, was confirmed as regulating two target genes, DNA replication licensing factor *MCM6* and monocarboxylate transporter *MCT1*, and shown to be important for the maintenance of *Wolbachia* density in mosquito cells^24^. However, a study of the function of miRNAs in the resistance mechanism of insects to *Bacillus thuringiensis* (Bt) toxins has not yet been published.

In the present study, we investigated and characterized the differential expression of miRNAs in the ACB larvae with different susceptibility to the Cry1Ab toxin using deep sequencing technology. The results will help to further understand the role of miRNAs in the ACB resistance to Bt toxins.

## Results

### Overview of the small RNA dataset

**Figure 1:**
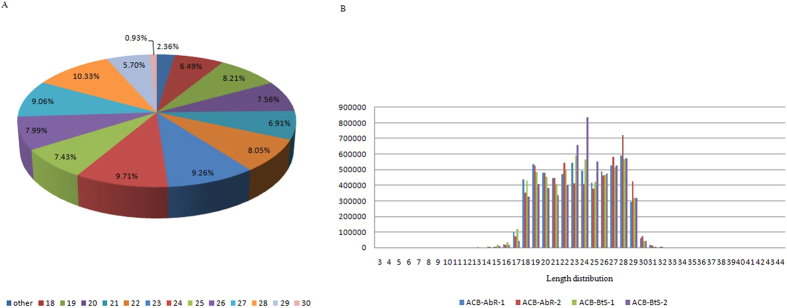
Size distribution of sRNA tags related to miRNAs in the four libraries of the Asian corn borer. (**A**) the size distribution of total sRNA, (**B**) the size distribution of each library.

**Figure 2:**
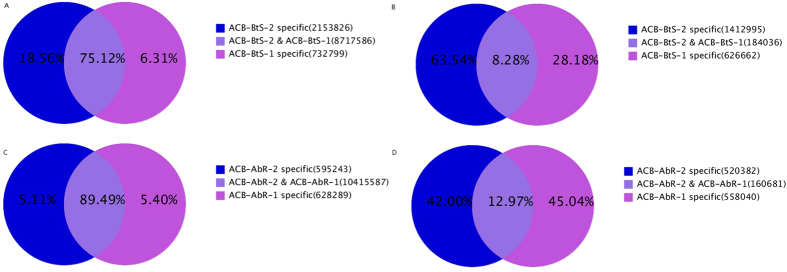
The common and specific tags in the two replicates of the Asian corn borer strains with different susceptibility to Cry1Ab (ACB-BtS and ACB-AbR). (**A**) summary of total tags between ACB-BtS-1 and ACB-BtS-2; (**B**) summary of unique tags between ACB-BtS-1 and ACB-BtS-2; (**C**) summary of total tags between ACB-AbR-1 and ACB- AbR-2; (**D**) summary of unique tags between ACB-AbR-1 and ACB-AbR-2.

Four small RNAs libraries, including two biological replicates of ACB-BtS and ACB-AbR, were constructed and Solexa-sequenced. A total of 23,809,890 high-quality reads were collected from the four libraries (Accession No: SRX976786) (Table 1). The reads with 5′ contaminants or without the 3′ primer or insert tag were removed, leaving 5,948,254 and 5,913,632 reads for the two ACB-BtS replicates and 5,941,222 and 5,937,261 reads for the ACB-AbR replicates for the sequence length distribution analysis (Fig. 1). Considering all four libraries combined, the majority (97.64%) of the sequences were 18–30 nucleotides long with a 21-to-23 nt group (24.22% of total reads) (Fig. 1), which is considered the standard size of miRNAs. Another type of RNA sequence found was 28–30 nt long, corresponding to pi-RNA-like sequences, and represented 16.96% of the total reads from the four libraries. Then, reads shorter than 18 nt or with poly A were discarded, leaving 5,763,509 and 5,840,702 clean reads (sRNAs) and 5,804,468 and 5,834,651 clean reads for each of the two replicates of ACB-BtS and ACB-AbR, respectively, for further analysis. Among them, 8,717,586 sRNAs were common tags of the two ACB-BtS samples, and 10,415,587 sRNAs were common tags of the two ACB-AbR samples (Fig. 2). Of the clean reads, 4,114,476 and 2,375,684 reads from the two replicates of ACB-BtS accounted for 71.39% and 40.67%, respectively, and 4,382,741 and 4,186,098 reads from the two replicates of ACB-AbR accounted for 75.51% and 71.75%, respectively, and were mapped to the ACB transcriptome (Supplemental File 1).

### Small RNA annotation

**Figure 3:**
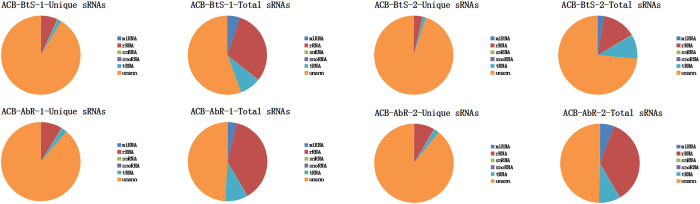
Composition of small RNA classes of the four libraries of the Asian corn borer.

During alignment and annotation, some small RNA tags may be mapped to more than one category. To have each unique small RNA map to only one annotation, we followed the priority rule of rRNAetc (in which GenBank > Rfam) > known miRNA > repeat > exon > intron^25^. The clean reads were divided into the following categories: miRNA, rRNA, snRNA, snoRNA, tRNA, and unann (sequences were not mapped to any known reference database). The composition of the RNA classes in each library is shown in Fig. 3. The proportion of total rRNA can be used as an indicator of sample quality. Typically, a proportion less than 60% in plant samples and 40% in animal samples indicates high quality (unpublished data by BGI)^26^. The proportions of rRNA in the total clean reads were 30.52% and 13.79% in the ACB-BtS-1 and ACB-BtS-2 samples and 38.05% and 35.80% in the ACB-AbR-1 and ACB-AbR-2 samples, indicating that the sequencing data were of high quality.

We identified 900 and 802 unique miRNA genes from the 297,610 and 150,645 total miRNAs in the two replicates of ACB-BtS and 816 and 915 unique miRNA genes from the 210,297 and 337,452 total miRNAs in the two replicates of ACB-AbR. However, due to the lack of a complete ACB genome database, the highest percentage of miRNA in the total sRNAs, which was found in ACB-AbR-2, was only 5.78%. A majority of the information remains unexplored in the datasets.

### Identification of known and novel miRNAs

To identify known miRNAs in the ACB, we searched the clean reads against the miRNA precursor/mature miRNAs of currently known miRNAs of all animals in the miRBase v21.0 database ( http://www.mirbase.org/). If the miRNA sequence with the most reads hit a known miRNA in miRBase, then the name of the known miRNA was assigned to the miRNA and other variants of the present study. In total, 302, 395, 268 and 287 known miRNAs were identified in ACB-BtS-1, ACB-BtS-2, ACB-AbR-1 and ACB-AbR-2, respectively. Among them, 163 miRNAs were commonly expressed in the four libraries (Supplemental File 2) of which the most abundant was Ofu-miR-1-3p, with an expression level reaching counts of 132,125, 70,521, 67,556 and 160,084 in the four libraries; the next most abundant miRNAs were Ofu-miR-6497–5p, Ofu-miR-11–5p, Ofu-miR-275–3p, and Ofu-miR-275. The 20 most abundant miRNAs expressed in the four libraries are listed in Table 2.

Using Mireap software, we identified 32, 16, 18 and 22 potential novel miRNAs in ACB-BtS-1, ACB-BtS-2, ACB-AbR-1 and ACB-AbR-2, respectively, with predicted precursor secondary structures (Supplemental File 3). Among them, 3 potential novel miRNAs were commonly expressed in the four libraries, and 4 potential novel miRNAs were commonly expressed in ACB-BtS-1, ACB-BtS-2, and ACB-AbR-1 (Table 3 and Supplemental File 4). A mature miRNA sequence mapped to different parts of the genome indicates that these gene loci represent different family members with either the same sequence or slightly different sequences that vary by only 1 or 2 nt. For example, Ofu-m0001, Ofu-m0002, Ofu-m0007, and Ofu-m0011 from ACB-AbR-1 were defined as novel miRNAs because they mapped to different locations in the ACB transcriptome despite having the same sequence (ATAAGTGGGAGATCGTTTCGGCG). The minimal free energy (MFE) of all potential novel miRNAs was below −18 kCal/mol, and their lengths ranged from 20 to 24 nt.

### Comparison of miRNA expression between ACB-BtS and ACB-AbR

**Figure 4:**
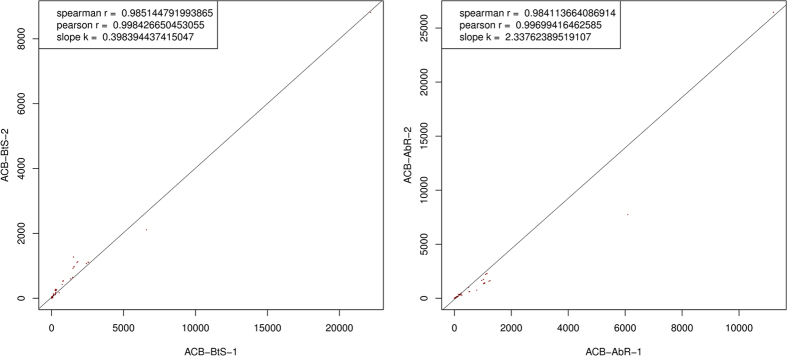
Pearson correlation analysis of replicates from Cry1Ab-susceptible (ACB-BtS) and -resistant (ACB-AbR) strains of the Asian corn borer.

**Figure 5:**
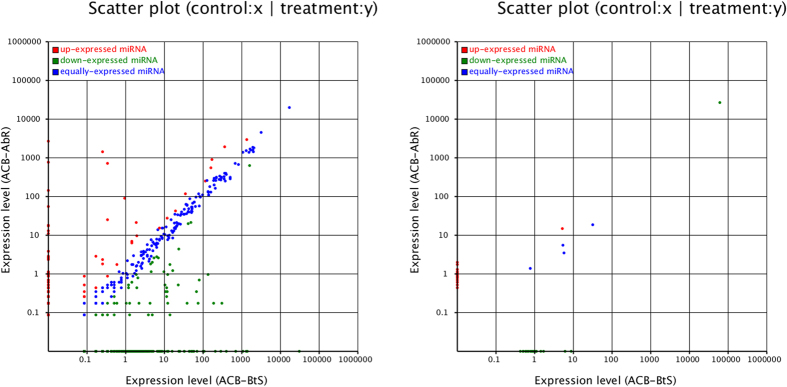
Expression of miRNAs in the Asian corn borer with differing susceptibility to Cry1Ab (ACB-BtS and ACB-AbR). (**A**) The differences of known miRNAs expression between ACB-BtS and ACB-AbR. (**B**) The differences of novel miRNAs expression between ACB-BtS and ACB-AbR. Note: The scatter plot of differentially expressed miRNAs (control: X-axis, treatment: Y-axis). The X and Y show the expression level of miRNAs in the two strains respectively. Red points represent miRNAs with ratio >2; Blue points represent miRNAs with 1/2< ratio ≤2; Green points represent miRNAs with ratio ≤1/2. Ratio = normalized expression of the treatment/normalized expression of the control.

The Pearson coefficient of all miRNAs in the two replicates of ACB-BtS and ACB-AbR (Fig. 4) indicated acceptable reproducibility. We normalized the expression of miRNAs in the four samples and calculated the fold-changes and *p*-values based on the normalized expression levels. Next, we generated a log_2_ratio figure and scatter plot (Fig. 5). According to the fold-change calculations, the expression levels of 555 known miRNAs and 45 novel miRNAs differed between ACB-BtS and ACB-AbR (Supplemental File 5). However, considering the *p*-values, only 21 known and 1 novel miRNAs (*p*-value < 0.05) were significantly differentially expressed between the two strains (Table 4). Among them, 10 known miRNAs and 1 novel miRNA showed higher expression levels in the ACB-AbR samples and 11 known miRNAs showed higher expression in the ACB-BtS samples.

**Figure 6:**
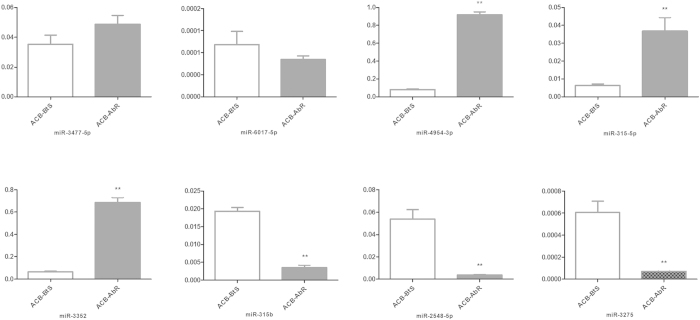
RT-qPCR validation of differentially expressed miRNAs identified in the Asian corn borer using Solexa sequencing technology. Note: **indicates the significant (p < 0.01) difference in expression level between Cry1Ab susceptible and resistant Asian corn borer by t-test of SAS software.

RT-qPCR was used to verify the expression levels of candidates differentially expressing miRNAs (Fig. 6). The results suggested that the expression levels of Ofu-miR-3477–5p, Ofu-miR-4954–3p, Ofu-miR-316–5p, and Ofu-miR-3352 were upregulated in ACB-AbR, whereas Ofu-miR-6017–5p, Ofu-miR-315b, Ofu-miR-2548–5p, and Ofu-miR-3275 were more highly expressed in ACB-BtS. The high confirmation rate indicated the reliability of our data.

### Target gene prediction for miRNAs

miRNAs work through imperfect base pairing to target seed region sites in the 3′UTR region of mRNAs, resulting in translational inhibition or mRNA degradation^27^. Using RNAhybrid software, 72,831 and 72,830 target genes were predicted for known miRNAs in ACB-BtS, and 72817 and 72815 target genes were predicted for known miRNAs in ACB-AbR. Additionally, 54,605 and 41,616 target genes were predicted for novel miRNAs in ACB-BtS, and 43,515 and 50,976 target genes were predicted for novel miRNAs in ACB-AbR (Supplemental File 6).

The ACB miRNAs were found to target several genes, including important enzymes, transcriptional factors, receptors and hormones. However, the focus of the present study was to gain insight into the mechanism of miRNAs regulating Bt resistance. Therefore, we analysed the miRNAs targeting candidate Bt receptor genes or other genes that are likely to be involved in insecticide resistance. miRNAs were observed to target potential Bt receptor genes, such as APN, CAD, ALP, and ABC transporter family protein. Moreover, several miRNAs were predicted to target Bt-related enzymes, such as trypsin-like enzyme, chymotrypsin-like enzyme, and carboxylesterase (CarE) (Table 5). Notably, some miRNAs were found to target to multiple genes, which was similar with the study in 2008^28^. For example, Ofu-miR-2731 was predicted to regulate the APN4 protein gene (CL9114.Contig1) and the ABC transporter B family protein gene (Unigene25984).

Analysis of the target genes of significantly differentially expressed miRNAs between ACB-BtS and ACB-AbR identified 65,301 and 4,925 target genes of known and novel differentially expressed miRNAs, respectively. When we focused on the target genes of the significantly differentially expressed miRNAs, the candidate Bt-related genes were also detected, such as APN, CAD, and ALP. Based on the transcriptome differences between ACB-BtS and ACB-AbR, the differential expression of selective Bt-related targeting miRNAs was compared with the transcript abundance of the target genes. Generally, if miRNA levels are low, their respective target levels are expected to be high, and *vice versa*. The results revealed that the expression of some of the miRNAs was negatively correlated with the relative abundance of their respective transcripts (Table 5). For example, the expression of Ofu-miR-3851c–5p was opposite that of its target miRNA APN1. However, the expression levels of CAD and ABC transporter family protein were in accordance with the expression of their respective miRNAs.

### Gene Ontology (GO) enrichment and KEGG pathway analysis of target genes

**Figure 7:**
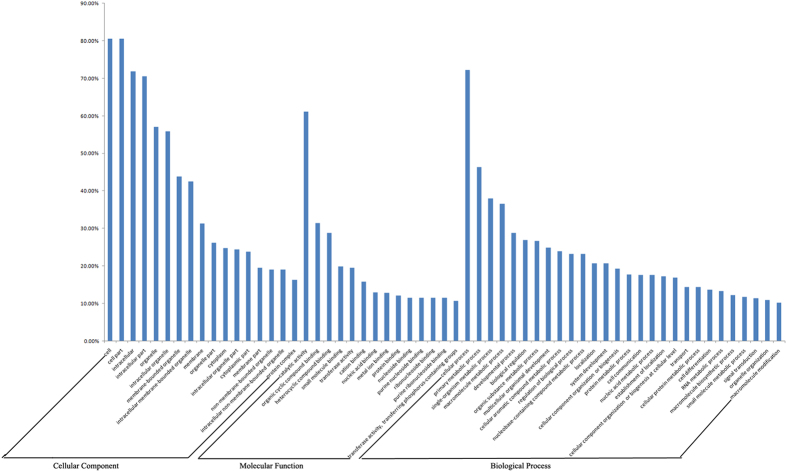
GO analysis of the most targeted genes of known differentially expressed miRNAs between Cry1Ab-susceptible and -resistant strains of the Asian corn borer (ACB-BtS and ACB-AbR).

**Figure 8:**
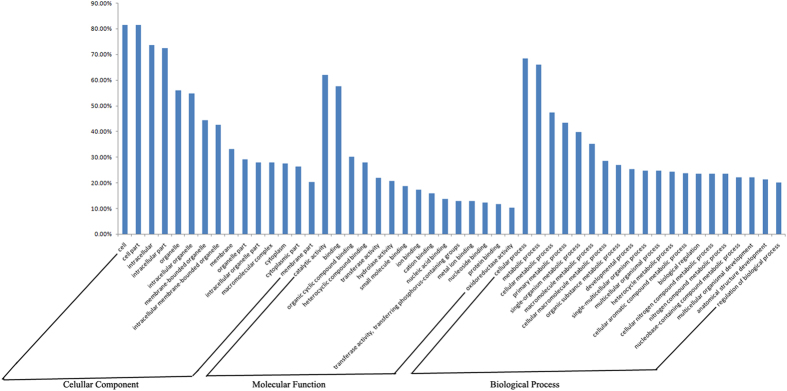
GO analysis of the most targeted genes of novel differentially expressed miRNAs between Cry1Ab-susceptible and -resistant strains of the Asian corn borer (ACB-BtS and ACB-AbR).

Through GO annotation, 6,515, 10,198, and 9,309 genes targeted to the known differentially expressed miRNAs were mapped to 324, 658, and 2,259 GO terms, respectively, corresponding to component, function and process ontology, respectively. In addition, 451, 648, and 637 genes targeted to the novel differentially expressed miRNAs were mapped to 143, 237, and 906 GO terms, respectively, in these areas (Supplemental File 7). The target genes of known differentially expressed miRNAs were largely involved in cell, cell part, catalytic activity, organic cyclic compound binding, heterocyclic compound binding, transferase activity, cellular process, primary metabolic process, macromolecule metabolic process, and developmental process (Fig. 7). These results suggest that miRNAs may affect the susceptibility of the ACB to Bt through regulation of the expression of the genes related to binding, transformation, metabolism and structure. In addition, the glycosylphosphatidylinositol (GPI) anchor biosynthetic process and GPI-anchor metabolic process were found through GO annotation of target genes, even though only 2 (Unigene 22631 and Unigene 5014) out of 9,303 genes were annotated to this GO term. Furthermore, GO analysis of the target genes of the novel differentially expressed miRNAs yielded similar results to those of the known miRNAs (Fig. 8).

**Figure 9:**
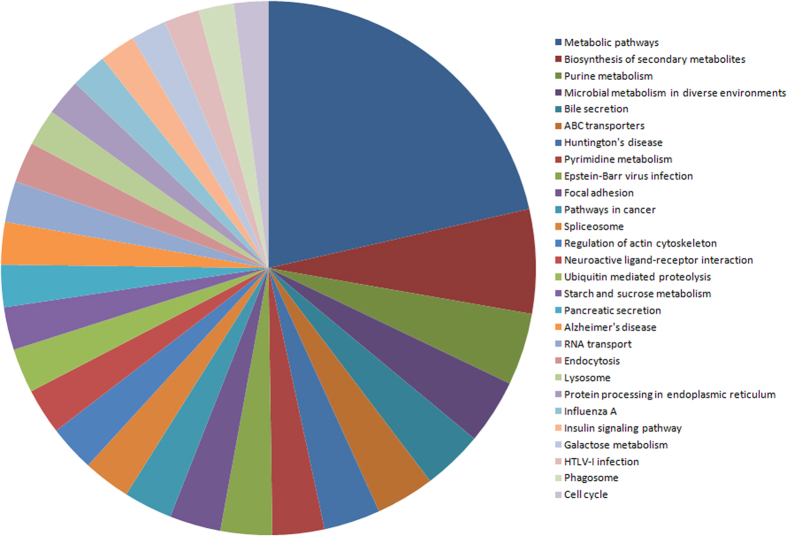
The number of target genes of known differentially expressed miRNAs between Cry1Ab-susceptible and -resistant strains of the Asian corn borer (ACB-BtS and ACB-AbR).

Based on the further analysis of biological pathways, 18,704 and 1,328 genes, targeted to the known and novel differentially expressed miRNAs, respectively, were mapped to 307 and 281 pathways (Supplemental File 8). Most of the target genes (20.62% and 23.04% in the known and novel differentially expressed miRNAs, respectively) were mapped to metabolic pathways (ko01100) (Fig. 9), which further illustrated that the miRNAs regulating the expression of metabolic genes play key roles in the Cry1Ab resistance of the ACB.

Other involved pathways were also informative. First, 6.05% and 6.4% target genes of known and novel differentially expressed miRNAs, respectively, were mapped to biosynthesis of secondary metabolites (ko01110) (Fig. 8). Second, 3.41% and 0.57% of known differentially expressed miRNAs and 4.44% and 0.9% of novel differentially expressed miRNAs were mapped to ABC transporters (ko02010) and the proteasome (ko03050), respectively. Third, 0.28% and 0.15% of known and novel differentially expressed miRNAs, respectively, were mapped to GPI-anchor biosynthesis (ko00563) (Supplemental File 8).

## Discussion

Insect resistance to *Bacillus thuringiensis* (Bt) is a major threat to the long-term use of Bt crops and has been studied extensively. However, understanding of the molecular mechanism of resistance of the ACB, the major corn insect pest in China and other countries of East and Southeast Asia, to Cry toxin is relatively poor. Over the last few years, studies have shown that miRNAs play a key role in host-pathogen interactions by regulating the expression of host resistant genes or viral genes to impair viral replication^29^,^30^. In this study, the small RNAs of two biological replicates of each of the ACB-BtS and ACB-AbR strains were sequenced by Illumina/Solexa technology. Then, the differentially expressed miRNAs between ACB-BtS and ACB-AbR were analysed, and the target genes of miRNAs were predicted. In addition, GO enrichment and KEGG pathway analyses were conducted based on the target genes of the differentially expressed miRNAs.

Since the discovery of the first miRNA from *Caenorhabditis elegans* over two decades ago^31^, a number of miRNAs have been identified in several insects using a variety of methods. Wu *et al.* used Illumina/Solexa deep sequencing technology to identify 168 known miRNAs belonging to 55 families as well as 204 novel miRNAs of fourth-instar larvae of male and female *Eupolyphaga sinensis* (Walker)^32^ Aided by next-generation sequencing and a microarray assay, Li *et al.* profiled 1,229 miRNAs of the posterior silk gland at the fifth larval instar^33^. Using Illumina, 32.9 million reads of 18,031 nucleotides were sequenced from four small RNA libraries using fat body and haemocytes from naïve and bacteria-injected larvae^34^. In the present study, approximately 23.8 million high-quality reads were collected from the ACB using Illumina/Solexa deep sequencing technology. This sequencing resulted in the identification of 302, 395, 268 and 287 known and 32, 16, 18, and 22 novel miRNA candidates from the ACB larvae with differing susceptibility to Cry1Ab. This is the first report on the ACB miRNAs. Two sequencing replicates of each strain were used, which strengthens the results, and the data enrich our knowledge of the ACB miRNAs.

A total of 163 known and 3 novel miRNAs were commonly expressed in the four libraries of the ACB. Few commonly expressed miRNAs candidates, such as miR-let-7, miR-bantam, and miR-14, have been reported to play essential roles in areas such as insect development^35^, innate immunity^36^, and metabolic defect^17^. The detection of classical miRNAs in the present study suggests that these conserved miRNAs may have important regulatory roles in the ACB.

According to the fold-changes, 555 known miRNAs and 45 novel miRNAs were differentially expressed between ACB-BtS and ACB-AbR. However, considering the *p*-values, only 21 known and 1 novel miRNAs were significantly differentially expressed between the two strains. Among them, miR-31 has been characterized as a tumour suppressor miRNA, with its level in cancer cells varying according to the metastatic state of the tumour^37^. Mse-miR-31 has been detected in *Manduca sexta* and was hypothesized to regulate evolutionarily conserved intermediate in Toll pathway (ECSIT), mitogen-activated protein kinase 1 (MEKK1), and Rel family protein 2B (Rel2B), which are involved in *M. sexta* immunity^34^. In *Plutella xylostella*, miR-31 was identified as one of the five most abundant miRNAs^38^. Twenty-eight miRNAs, including miR-31, were identified as differentially expressed between deltamethrin-sensitive and -resistant strains of mosquitoes^39^. These differentially expressed miRNAs might be involved in the differential susceptibility of the ACB to Cry1Ab.

The target genes of miRNAs in the ACB were predicted using the transcriptome of the ACB as a reference. miRNAs were found to target several important candidate Bt receptor genes or other genes that are likely to be involved in insecticide resistance in insects: APN, CAD, ALP, ABC transporter family protein, trypsin-like enzyme, chymotrypsin-like enzyme, CarE, and AChE. Different isoforms of APNs and CAD together with ALP have been shown to interact with different types of Cry toxins^40^. It has been reported that P450, CaE, GST, superoxide dismutase (SOD), and prophenoloxidase (PPO) are related to the metabolism of the insecticide. The activity of CarE was higher in ACB-AbR than in ACB-BtS; although no significant difference was detected in AchE between the two strains^41^.

The prediction of putative target transcripts of differentially expressed miRNAs between ACB-BtS and ACB-AbR helps reveal the post-transcriptional regulation of gene expression in ACB with resistance to Cry1Ab. We predicted the target genes of 21 significantly differentially expressed miRNAs. A large number of target genes were predicted from the transcriptome data of the ACB. Considering the results of transcriptome differences between ACB-BtS and ACB-AbR, the expression of some miRNAs and their respective targets were negatively correlated. For example, the expression of APN1 (CL3709.Contig1), APN2 (Unigene9047), APN3 (Unigene33230), and APN4 (CL9114.Contig1) was upregulated in ACB-AbR^7^. The expression of Ofu-miR-3851c–5p, Ofu-miR-963–3p, Ofu-miR-927–3p, and Ofu-miR-2731, which were predicted to target the respective genes, were downregulated in ACB-AbR. Similar trends were also detected in the expression of Ofu-miR-6038, Trypsin-like serine protease (CL2723.Contig2), Ofu-miR-3897–3p and Chymotrypsin-like protease (Unigene36951). However, not all expression of miRNAs was negatively related to the target genes. The expression Ofu-miR-6012–5p was shown to be downregulated in ACB-AbR, whereas its target, cadherin-like protein gene (Unigene24705), was also decreased in ACB-AbR. However, the expression of Ofu-miR-316–5p and Ofu-miR-3250, which were also predicted to target this gene, was increased in ACB-AbR, although with *p*-values >0.1. It has been shown that several miRNAs have multiple target genes and that numerous genes have mutually common targeting miRNAs^42^. We assume that some gene is regulated by multiple miRNAs to influence the susceptibility of the ACB to Cry1Ab.

GO annotation and KEGG pathway analyses provide a better understanding of the cellular components, molecular functions and biological processes of target genes^43^. The predicted target genes were classified into different functional categories according to GO annotation. The majority of the predicted target genes were involved in cell, cell part, catalytic activity, binding, transferase activity, cellular process, metabolic process, and developmental process, similar to the *Wolbachia*-responsive miRNAs in *T. urticae*^22^. The KEGG pathway analysis revealed that metabolic pathways (ko01100), biosynthesis of secondary metabolites (ko01110), purine metabolism (ko00230), and microbial metabolism in diverse environments (ko01120) were the top four pathways with the most target genes of differentially expressed miRNAs between ACB-BtS and ACB-AbR. This result implies that the resistance mechanism associated with metabolism is important in the resistance of *O. furnacalis* to Cry1Ab.

In addition, 3.41% of the target genes of known differentially expressed miRNAs were mapped to ABC transporters (ko02010) that have been identified as related to Bt resistance^44^,^45^. A strain of *B. mori* that was resistant to Cry1Ab toxin also revealed a mutation in a homologous ABC transporter^46^. In addition, the expression changes of ABC transporter have been reported to contribute to Bt resistance. In the transcriptome of *P. xylostella*, eight unigenes from ABCC2 were detected in the Cry1Ac-resistant strain, and a majority of them were downregulated^47^. In the transcriptome of ACB, nine unigens, enriched to ACB transporters, were differentially expressed between ACB-BtS and ACB-AbR, four of which were up-regulated and five down-regulated in ACB-AbR^48^.

In addition, the GPI-anchor metabolic/biosynthetic process and GPI anchor biosynthetic pathway (ko00563) were both detected in GO annotation and KEGG pathway analysis. GPI-anchored proteins such as APN and ALP have been identified as Cry-toxin-binding receptors in Lepidoptera. An *H. virescens* population that underwent selection in the laboratory showed a lack of ALP^49^. A *Spodoptera exigua* strain resistant to Cry1Ca and a *Trichoplusia ni* strain resistant to Cry1Ac also showed reduced APN transcripts^50^. A laboratory-selected *Helicoverpa armigera* strain with Cry1Ac resistance was found to contain a deletion mutation in APN^51^. GPI-anchored receptors have been found to play an important role in membrane insertion and pore formation^52^. Cry-toxin binding to GPI-anchored receptors in *M. sexta* was found to actuate the localization of APN and ALP in lipid raft micro domains where Cry toxin inserts and forms pores^51^. Eighteen differentially expressed genes between ACB-BtS and ACB-AbR were found to be involved in GPI-anchor biosynthesis, and all genes mapped to the pathway were substantially down-regulated (6.6 to 13.0 times) in ACB-AbR^48^. We speculate that the miRNAs related to the GPI-anchor biosynthesis process are important in the Cry1Ab resistance of the ACB.

In summary, this is the first report identifying the ACB miRNAs. The study reveals new information on the ACB miRNAs that are associated with Cry1Ab resistance and may be helpful in studying Bt resistance mechanisms in other Lepidoptera insects. Further function experiments are necessary to elucidate the underlying mechanism of Bt resistance in the ACB.

## Materials and Methods

### Asian corn borer strains and sample preparation

The laboratory strain of the ACB was originally collected from a summer corn field in central China. It was maintained at 27±1°C, 70–80% relative humidity (RH) and a 16:8 (L:D) photoperiod at the Institute of Plant Protection, Chinese Academy of Agricultural Sciences, Beijing. During this period, the strain had no contact with any insecticides. This strain was considered to be a susceptible strain (designated ACB-BtS). Based on the ACB-BtS strain, trypsin-activated Cry1Ab toxin (94% pure protein) was used as a source of Cry1Ab for the selection diet. The selection treatment strain (ACB-AbR) was initially exposed throughout larval development to Cry1Ab in an artificial diet (2.5 ng toxin/g). The toxin concentration was steadily increased in succeeding generations to target 40–70% mortality in the exposed insects. After 51 generations, the ACB-AbR strain was reared on a diet containing 400 ng toxin/g. Previous work found that ACB-AbR developed more than 100-fold resistance to Cry1Ab after 35 generations of selection^6^. After more than 135 generations of selection, the ACB-AbR strain was used to detect the potential miRNAs associated with Cry1Ab resistance in ACBs, whereas the ACB-BtS strain, which was reared in the absence of any toxin, was used as the negative control strain. Five larvae, one individual larva from each instar from 1–5 instar larvae, were placed in a PE tube as one biological replication for each of the ACB-BtS and ACB-AbR strains. Five biological replicates of each sample were collected and processed independently. Two replicates were used for microRNA expression profile analysis, and the others were used for the RT-qPCR analysis. All samples were stored at -80°C until assayed.

### Small RNA library construction and sequencing

Total RNAs, each from two biological replicates of ACB-BtS and ACB-AbR, were extracted using TRIzol reagent (Invitrogen, Carlsbad, CA, USA). Separated on 15% denaturing polyacrylamide gel, small RNA fragments in the 18–30 nt range were purified, and then 3′ and 5′ RNA adaptors were ligated to the RNA pool using T4 RNA ligase. These fragments were then used for reverse transcription and subsequent PCR. The final PCR products were purified and subjected to the proprietary Solexa sequencing-by-synthesis method using the Illumina Genome Analyzer (San Diego, CA, USA) at the Beijing Genomics Institute (BGI, Shenzhen, China).

### Bioinformatic analysis of Solexa sequencing data

The sequences from the HiSeq sequencing were deposited in the NCBI Sequence Read Archive (SRA). The reads under 50 nt sequence length were first subjected to data cleaning, which included the removal of low-quality reads, reads with 5′ primer contaminants, reads without 3′ primers and reads without the insert tag. The length distribution of the remaining sRNA was then summarized. Then, reads with either poly (A) or shorter than 18 nt were removed, and the final clean reads, which were assigned to two groups including the summary of unique reads and total reads, were used for standard bioinformatics analysis. The common and specific reads of the two groups were summarized, including summaries of unique reads and total reads. As data on the ACB genome are not yet available, the clean reads of the ACB-BtS and ACB-AbR libraries were mapped to the ACB transcript database (Accession No: SRP046207) using SOAP software v1.11 to analyse sRNA expression and distribution in the genome. The clean reads were annotated into different categories to discard rRNAs, tRNAs, snRNAs, and snoRNA using Rfam database v10.1. Because there was no information concerning the miRNAs of the ACB in the miRBase v21.0, the remaining sRNAs were aligned to the miRNA precursors/mature miRNAs of all animals in the miRBase to detect the sequence and count of miRNA families (no specific species) in the samples.

The characteristic hairpin structure of miRNA precursors can be used to predict novel miRNA candidates. Due to the lack of genome information for the ACB, the genome sequences of *Ostrinia scapulalis* (Accession No: PRJNA192419) were used as a reference for novel miRNA prediction. The prediction software Mireap (http://sourceforge.net/projects/mireap/) was used to predict novel miRNA candidates by exploring the secondary structure, the Dicer cleavage site, and the minimum free energy of the unannotated small RNA reads that could be mapped to the genome.

### Differences in miRNA expression between ACB-BtS and ACB-AbR

The expression of miRNAs was compared between ACB-BtS and ACB-AbR to identify differentially expressed miRNAs. First, the expression of miRNA in the four libraries was normalized to transcripts per million (TPM). If the normalized expression of the miRNA was 0, it was modified to 0.01 to enable calculation. If the normalized expression of the miRNA was less than 1 in all libraries, it was ignored to compare for low expression. The normalization formula was: Normalized expression = Actual miRNA count/Total count of clean reads × 10^6^. Second, Pearson correlation analysis was conducted to test the relativity between the two replicates of ACB-BtS and ACB-AbR. A Pearson coefficient for miRNA expression between replicates of more than 0.85 indicates a strong correlation between the replicates. The normalized data were then used to calculate fold-change values and *P*-values, and a scatter plot of the fold-change values was generated. Fold-change was calculated as Fold-change = log_2_(ACB-AbR/ACB-BtS). The *P*-value was calculated through equation 1:


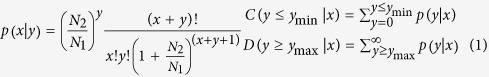


where *x* represents ACB-BtS, *y* represents ACB-AbR, *N*_*1*_ represents the normalized expression of a miRNA in ACB-BtS library, and *N*_*2*_ represents the normalized expression of the same miRNA in ACB-AbR library.

### Verification of miRNAs by quantitative real-time PCR (qRT-PCR)

To validate the relative expression levels of the identified miRNAs in our libraries, we selected 8 differentially expressed miRNAs for qRT-PCR. Total RNA was extracted from the same samples used for deep sequencing. The specific forward primers of 8 selected miRNAs were designed according to the sequence of each miRNA itself and are provided in Supplemental File 9. The reverse transcription reaction was performed with the First Strand cDNA Synthesis Kit (Thermo Fisher, USA) according to the manufacturer’s protocol. qRT-PCR was performed with the SYBRGreen PCR kit (Thermo Fisher, USA) on an ABI-7300 machine (Applied Biosystems, USA) with thermal cycling parameters of 95 °C for 10 min, followed by 40 cycles at 95 °C for 15 s, and then 60 °C for 45 s, according to the manufacturer’s protocol. Three biological replicates, each with three technical replicates, were used for qRT-PCR, and U6 snRNA was used as an internal reference. The threshold cycle (CT) was collected from each reaction, and the relative expression level of each miRNA to U6 snRNA was evaluated using the equation 2^−(CTmiRNA-CTU6RNA)^. A t-test was used to examine the significance of expression differences between the two samples using SAS v8.0 software.

### Target prediction

The putative target sites of miRNA candidates were identified by aligning the miRNA sequences with the ACB transcriptome and other published gene data from Lepidoptera insects using the RNAhybrid software ( http://bibiserv.techfak.uni-bielefeld.de/rnahybrid/). The rules used for target prediction were based on those suggested by Allen *et al.* and Schwab *et al.*^53^,^54^. The target genes of the known miRNAs, novel miRNAs, and differentially expressed miRNAs between ACB-BtS and ACB-AbR were predicted.

### GO enrichment and KEGG pathway analysis

To further analyse the function of differentially expressed miRNAs between ACB-BtS and ACB-AbR, Gene Ontology (GO) and the Kyoto Encyclopedia of Genes and Genomes (KEGG) pathway analysis were used on predicted target gene candidates of differentially expressed miRNAs. GO is an international standardized classification system for gene function, which supplies a set of controlled vocabulary to comprehensively describe the property of genes and gene products. There are 3 ontologies in GO: molecular function, cellular component and biological process. The GO results were used to reveal the functions significantly related with predicted target gene candidates of the detected miRNAs. First, all target gene candidates were mapped to GO terms in the database ( http://www.geneontology.org/), calculating gene numbers for each term. Then, a hypergenometric test was used to find GO terms in target gene candidates that were significantly enriched compared with the reference gene background. The calculation used for the test was equation 2:


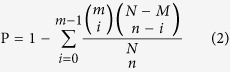


where *N* is the number of all genes with GO annotation, *n* is the number of target gene candidates in *N, M* is the number of all genes that were annotated to a specific GO term, and *m* is the number of target gene candidates in *M*. The Bonferroni correction was used to obtain corrected *p*-values. GO terms with corrected *p*-values ≤0.05 were defined as significantly enriched in target gene candidates. This analysis recognizes the main biological functions of target gene candidates.

KEGG is the major public pathway-related database. In organisms, genes usually interact with each other to play different roles in specific biological functions. KEGG pathway analysis can facilitate understanding of the biological functions of genes. KEGG pathway analysis identifies significantly enriched metabolic pathways or signal transduction pathways in target gene candidates through comparison with the entire reference gene background. The formula used was the same as that in GO analysis. Here, *N* is the number of all genes with KEGG annotation, *n* is the number of target gene candidates in *N, M* is the number of all genes annotated to a specific pathway, and *m* is the number of target gene candidates in *M*. Genes with FDR ≤0.05 were considered significantly enriched in target gene candidates. The KEGG analysis revealed the main pathways that the target gene candidates are involved in.

## Additional Information

**How to cite this article**: Xu, L.-N. *et al.* Identification of differentially expressed microRNAs between *Bacillus thuringiensis* Cry1Ab-resistant and -susceptible strains of *Ostrinia furnacalis. Sci. Rep.* **5**, 15461; doi: 10.1038/srep15461 (2015).

## Supplementary Material

Supplementary Information

Supplementary Information

Supplementary Information

Supplementary Information

## Figures and Tables

**Table 1:** The classification of total small RNA tags of the Asian corn borer by Solexa sequencing.

**Type**	**ACB-BtS-1**		**ACB-BtS-2**		**ACB-AbR-1**		**ACB-AbR-2**	
	**Counts**	**Percent**	**Counts**	**Percent**	**Counts**	**Percent**	**Counts**	**Percent**
High quality reads	5,964,851	100%	5,939,238	100%	5,953,504	100%	5,952,297	100%
3′ adapter-null	4,147	0.07%	14,176	0.24%	3,907	0.07%	4,433	0.07%
Insert-null	1,228	0.02%	864	0.01%	3,018	0.05%	1,395	0.02%
5′ adapter-contaminants	11,222	0.19%	10,566	0.18%	5,357	0.09%	9,208	0.15%
Smaller-than-18nt	184,717	3.10%	72,912	1.23%	136,741	2.30%	102,600	1.72%
PolyA	28	0.00%	18	0.00%	13	0.00%	10	0.00%
Clean reads	5,763,509	96.62%	5,840,702	98.34%	5,804,468	97.50%	5,834,651	98.02%

**Table 2:** The 20 most abundant miRNAs commonly expressed in the four libraries of the Asian corn borer.

**miRNA**	**Sequence**	**Counts**			
		**ACB-BtS-1**	**ACB-BtS-2**	**ACB-AbR-1**	**ACB-AbR-2**
Ofu-miR-1–3p	TGGAATGTAAAGAAGTATGGAG	132125	70521	67556	160084
Ofu-miR-6497–5p	TCTGAGGACCGGGGCGTGT	27407	9782	21997	29811
Ofu-miR-11–5p	AGAACTCCGGCTTACTCGAACTGTG	11150	4663	17199	16649
Ofu-miR-275–3p	TCAGGTACCTGAAGTAGCGCGCG	15368	8790	7024	14004
Ofu-miR-275	TCAGGTACCTGAAGTAGCGCG	15604	6943	7301	14375
Ofu-miR-2766–3p	TCAGTCTTGTCGAATGGTG	10961	11683	7626	10628
Ofu-miR-2816	CTCAGTGAGGATGGAGCGT	16877	6472	8223	8138
Ofu-miR-184	TGGACGGAGAACTGATAAGGG	11731	7391	8450	10998
Ofu-miR-184–3p	TGGACGGAGAACTGATAAGGGC	11350	8694	7966	10309
Ofu-miR-3389–3p	CGCCTGGGAACACCGCGTG	11379	6756	7500	8387
Ofu-let-7–5p	TGAGGTAGTAGGTTGTATAG	8816	6480	6467	11176
Ofu-let-7	TGAGGTAGTAGGTTGTATAGTA	8453	3951	5934	10162
Ofu-miR-6497–3p	GGAGGCGGCCGGTGCCGGGC	8948	9981	3845	3460
Ofu-miR-3262	AGGGCTCTGGAATAGTTGAAGA	2	4253	11312	10562
Ofu-miR-263a–5p	AATGGCACTGGAAGAATTCACGG	5082	4468	3500	4138
Ofu-miR-2766	TCAGTCTTGTCGAATGGTTT	4345	3707	3558	4620
Ofu-miR-4981–3p	TTGTGCTCGGTAGAGCAGCGTCGTG	1334	666	5369	4875
Ofu-miR-210	CGGTGGTAGTGACAACGG	1592	4344	1501	2067
Ofu-miR-6496—5p	ATAGCCCAGCACTGAATCCCGCGGT	3482	2429	1918	1396
Ofu-miR-8–3p	TAATACTGTCAGGTAAAGATGTC	2546	2013	2134	2454

**Table 3:** The potential novel miRNAs commonly expressed in the four libraries of the Asian corn borer.

**ACB-BtS-1**	**Name**							
	**ACB-BtS-2**	**ACB-AbR-1**	**ACB-AbR-2**	**Sequence**	**Length (nt)**	**Location**	**Precursor Length (nt)**	**Engergy kcal mol**^**−1**^
Ofu-m0012_5p	Ofu-m0003_5p	Ofu-m0004_5p	Ofu-m0001_5p	AGATCCGGCTCGAAGGACCA	20	CL4356.Contig2	78	−22.66
Ofu-m0015_3p	Ofu-m0005_3p	Ofu-m0006_3p	Ofu-m0008_3p	TGGAACATGAGCACTGGGGAAGT	23	CL9226.Contig1	79	−19.1
Ofu-m0021_5p	Ofu-m0011_5p	Ofu-m0010_5p	Ofu-m0013_5p	ACTTCGAACAGCAGCGAGACAT	22	Unigene15810	95	−22
Ofu-m0007_5p	Ofu-m0001_5p	Ofu-m0001_5p	Non	ATAAGTGGGAGATCGTTTCGGCG	23	CL192.Contig5	79	−21.1
Ofu-m0008_5p	Ofu-m0002_5p	Ofu-m0002_5p	Non	ATAAGTGGGAGATCGTTTCGGCG	23	CL192.Contig8	79	−21.1
Ofu-m0016_5p	Ofu-m0007_5p	Ofu-m0007_5p	Non	ATAAGTGGGAGATCGTTTCGGCG	23	GAHQ01004236.1	79	−21.1
Ofu-m0022_5p	Ofu-m0012_5p	Ofu-m0011_5p	Non	ATAAGTGGGAGATCGTTTCGGCG	23	Unigene18583	79	−21.1
Ofu-m0030_5p	Non	Ofu-m0017_5p	Ofu-m0021_5p	GTAGCTTGGGGTCATTGGACA	21	Unigene3306	75	−20.8
Non	Ofu-m0013_5p	Non	Ofu-m0018_5p	CGATGTCACACTGTCGTCGCA	21	Unigene19012	81	−49.2
Non	Non	Ofu-m0018_5p	Ofu-m0022_5p	GCTATTTGTGGTCTGCTACATTC	23	Unigene5004	84	−23.7

Note: Non means the specific miRNA was not predicted in the sample.

**Table 4:** The different expression of miRNAs between the Asian corn borer with differing susceptibility to Cry1Ab (ACB-BtS and ACB-AbR).

**miR-name**	**ACB-AbR-expressed**	**ACB-BtS-expressed**	**ACB-AbR-std**	**ACB-BtS-std**	**fold-change (log2 ACB-AbR/ACB-BtS)**	***p*****-value**
Ofu-miR-4954–3p	31180	0	2678.8969	0.01	18.0313	2.73E–02
Ofu-miR-2859	30	0	2.5775	0.01	8.0098	3.69E–02
Ofu-miR-2833b	10	0	0.8592	0.01	6.4249	3.86E–02
Ofu-miR-1a–5p	8	0	0.6873	0.01	6.1029	6.72E–06
Ofu-miR-3737	5	0	0.4296	0.01	5.4249	3.86E–02
Ofu-miR-3271	4	0	0.3437	0.01	5.1029	6.72E–06
Ofu-miR-184–5p	2	0	0.1718	0.01	4.1029	6.72E–06
Ofu-miR-31	2	0	0.1718	0.01	4.1029	6.72E–06
Ofu-miR-4981–3p	10244	2000	880.1353	172.3512	2.3524	1.07E–02
Ofu-miR-2745	3840	3168	329.9219	273.0043	0.2732	1.32E–02
Ofu-miR-306a–3p	16	31	1.3747	2.6714	−0.9585	2.24E–02
Ofu-miR-6497–3p	7305	18929	627.6248	1631.2182	−1.3780	7.19E–03
Ofu-miR-6038	0	2	0.01	0.1724	−4.1073	4.42E–05
Ofu-miR-6012–5p	0	4	0.01	0.3447	−5.1073	4.42E–05
Ofu-miR-963–3p	0	5	0.01	0.4309	−5.4292	4.01E–02
Ofu-miR-927–3p	6	268	0.5155	23.0951	−5.4855	2.07E–02
Ofu-miR-193–5p	1	45	0.0859	3.8779	−5.4962	4.25E–03
Ofu-miR-2731	0	21	0.01	1.8097	−7.4996	2.16E–02
Ofu-miR-3897–3p	2	454	0.1718	39.1237	−7.8309	6.65E–03
Ofu-miR-3851c–5p	0	30	0.01	2.5853	−8.0142	5.33E–03
Ofu-miR-2944a–5p	0	61	0.01	5.2567	−9.0380	3.10E–03
Ofu-novel-mir-14	15	0	1.2338	0.01	7.0098	3.86E–02

**Table 5:** The proteases and related target genes of miRNAs from the Asian corn borer.

**Unigene ID**	**Annotation**	**Log**_**2**_**Ratio**	***Q*****-value**	**microRNA**	**Fold change**	***p*****-value**
CL3709.Contig1	Aminopeptidase N1	3.18	0.7736	Ofu-miR-3851c–5p	−8.0142	0.005328
				Ofu-miR-2780d	0.1477	0.8921
Unigene9047	Aminopeptidase N2	2.78	0.8334	Ofu-miR-963–3p	−5.4292	0.04005
				Ofu-miR-998–5p	4.1029	0.4226
Unigene33230	Aminopeptidase N3	2.47	0.8042	Ofu-miR-927–3p	−5.4855	0.02071
				Ofu-miR-3387–5p	1.9957	0.3145
CL9114.Contig1	Aminopeptidase N4	0.098	0.1367	Ofu-miR-2731	−7.4996	0.02159
				Ofu-miR-3885–5p	−2.0588	0.4739
Unigene11733	Aminopeptidase N5	—		Ofu-miR-2941	10.8034	0.4226
Unigene12395	Aminopeptidase N7	—		Ofu-miR-4917–3p		
CL4275.Contig2	Aminopeptidase N8	—		Ofu-miR-279b	−0.3898	0.7886
CL9174.Contig1	Aminopeptidase N9	—		Ofu-miR-2780a–5p	5.9103	0.4226
Unigene24705	Cadherin-like protein gene	−12.10	0.8875	Ofu-miR-6012–5p	−5.1072	4.4248E-05
				Ofu-miR-316–5p	0.5806	0.8106
CL1041.Contig10	Alkaline phosphatase	—		Ofu-miR-3250	6.6879	0.4226
Unigene25984	ABC transporter family protein	−8.32	0.9166	Ofu-miR-2731	−7.4996	0.02159
				Ofu-miR-279b	−0.3898	0.7886
CL2723.Contig2	Trypsin-like serine protease	1.82	0.7500	Ofu-miR-6038	−4.1073	4.4248E-05
				Ofu-miR-2941	10.8034	0.4226
Unigene36951	Chymotrypsin-like protease	2.80	0.8373	Ofu-miR-3897–3p	−7.8309	0.006654
				Ofu-miR-184–5p	4.1029	6.7248E-06
CL951.Contig2	Glutathione S-transferase	−3.02	0.8346	Ofu-miR-193–5p	−5.4962	0.004254
				Ofu-miR-998–5p	4.1029	0.4226
CL2256.Contig1	Cytochrome P450	−6.78	0.8737	Ofu-miR-2944a–5p	−9.0380	0.003101
				Ofu-miR-2780a–5p	5.9103	0.4226
CL6671.Contig1	Carboxylesterase	5.18	0.8951	Ofu-miR-193–5p	−5.4962	0.004254

Note: Log_2_Ratio indicated the expression difference of unigene which were detected in the study of transcriptome differences between Cry1Ab resistant and susceptible strains of Asian corn borer (data were in publishing), and – means there was no difference of the unigene between the two strains.
