# Supplementary material for: Identification of differentially expressed microRNAs between Bacillus thuringiensis Cry1Ab-resistant and -susceptible strains of Ostrinia furnacalis
Source: Sci Rep. 2015 Oct 21;5:15461. doi: 10.1038/srep15461 (PMC4614346; doi:10.1038/srep15461)
Supplement: Supplementary Information [file srep15461-s1.pdf]

SUPPLEMENTARY INFORMATION

Identification of differentially expressed microRNAs between *Bacillus thuringiensis* Cry1Ab-resistant and -susceptible strains of *Ostrinia furnacalis*

Li-Na Xu[1, 2†], Ying-Hui Ling[3†], Yue-Qin Wang[2], Zhen-Ying Wang[2], Ben-Jin Hu[1], Zi-Yan Zhou[1], Fei Hu[1], Kang-Lai He[2*]

1 Institute of Plant Protection and Agro-Products Safety, Anhui Academy of Agricultural Sciences, Hefei, Anhui 230031, China

2 The State Key Laboratory for Biology of Plant Diseases and Insect Pests, Institute of Plant Protection, Chinese Academy of Agricultural Sciences, Beijing 100193, China

3 College of Animal Science and Technology, Anhui Agricultural University, Hefei, Anhui 230036, China

* Corresponding author: Kang-Lai He

Email: hekanglai@caas.cn

Tel: +86-10-62815932

Fax: +86-10-62815932

Supplemental File 1: The mapping statistics of sRNAs (Table S1)

Supplemental File 2: The known miRNAs (Table S2)

Supplemental File 3: The potential novel miRNAs (Table S3)

Supplemental File 4: The common expressed novel miRNAs (Table S4)

Supplemental File 5: The different expression of miRNAs (Table S5-6, included as separate Excel documents)

Supplemental File 6: The statistic of target gene of miRNAs (Table S7)

Supplemental File 7: The GO enrichment analysis of differentially expressed miRNAs (Table S8-13, included as separate Excel documents)

Supplemental File 8: The KEGG pathway analysis of differentially expressed miRNA (Table S14-15, included as separate Excel documents)

Supplemental File 9: The primers used in the study for quantitative real-time PCR (Table S16)

Table S1 The mapping statistics of sRNAs from four libraries of Asian corn borer

	ACB-BtS-1		ACB-BtS-2		ACB-AbR-1		ACB-AbR-2	
	Total sRNAs	Mapping to transcriptome	Total sRNAs	Mapping to transcriptome	Total sRNAs	Mapping to transcriptome	Total sRNAs	Mapping to transcriptome
Unique sRNAs	810,698	401,262	1,597,031	326,610	718,721	420,078	681,063	391,588
Percent(%)	100%	49.50%	100%	20.45%	100%	58.45%	100%	57.50%
Total sRNAs	5,763,509	4,114,476	5,840,702	2,375,684	5,804,468	4,382,741	5,834,651	4,186,098
Percent(%)	100%	71.39%	100%	40.67%	100%	75.51%	100%	71.75%

Table S2 The co-expressed miRNAs in the four libraries of Asian corn borer

		ACB-BtS-1	ACB-BtS-2	ACB-AbR-1	ACB-AbR-2
MiRNA	Sequence	Counts	Counts	Counts	Counts
miR-iab-4-5p	ACGTATACTGAATGTATCCTGA	36	39	39	33
miR-iab-4-3p	CGGTATACCTTCAGTATACGT	7	7	2	4
miR-9d-3p	ATAAAGCTAGATTACCAA	51	2	28	59
miR-9c-5p	TCTTTGGTATCCTAGCTGTAG	329	276	172	384
miR-9c-3p	TAAAGTTATGGTACCGAAGTTA	136	108	100	96
miR-9b-3p	ATAAAGCTAGATTACCAAAGCA	48	41	28	58
miR-9a-5p	TCTTTGGTTATCTAGCTGTATGA	533	18	230	414
miR-9a-3p	TAAAGCTAGGTTACCGGAGTTA	108	84	57	79
miR-998-3p	TAGCACCATGGGATTCAGCTCA	42	30	19	36
miR-998	TAGCACCATGGGATTCAGCT	46	34	25	45
miR-993b-5p	TACCCTGTAGATCCGGGCTTT	110	103	120	119
miR-993-5p	CTACCCTGTAGATCCGGGCTTTTG	86	17	85	88

miR-993-3p	GAAGCTCGTCTCTACAGGTATCT	33	34	23	44
miR-993	TACCCTGTAGATCCGGGCTTTT	108	138	121	121
miR-989a	GTGTGATGTGACGTAGTGGAA	214	104	180	216
miR-989	GTGTGATGTGACGTAGTGGAAG	213	154	181	218
miR-970-3p	TCATAAGACACACGCGGCTCT	1642	1439	1154	1995
miR-965-5p	CGGGAGAAGCTGTATCGCTATATG	610	536	356	903
miR-965-3p	TAAGCGTATAGCTTTTCCCCTT	16	15	13	8
miR-932-3p	TGCAAGCGCTGGTGGACTTCA	5	3	6	7
miR-92b-3p	AATTGCATTAATCCCGGCCTG	1	5	1	1
miR-92b	AATTGCACCAATCCCGGCCTG	75	78	34	64
miR-92a	TATTGCACCAGTCCCGGCCTAT	128	82	86	91
miR-928	CGCTGTGGAAGTTGGCAA	352	247	357	251
miR-927-3p	AAAGCTTTTGGATTCATAC	114	154	2	4
miR-87	GTGAGCAAACTTTCAGGTGTGT	8	12	5	7
miR-8-5p	CATCTTACCGGGCAGCATTAGA	2128	2066	1583	3043
miR-8-3p	TAATACTGTCAGGTAAAGATGTC	2546	2013	2134	2454
miR-79-3p	TAAAGCTAGATTACCAAAGCAA	43	17	24	52
miR-750-3p	CCAGATCTATCTTTCCAGCT	321	157	236	230
miR-750	CCAGATCTATCTTTCCAGCTCA	308	198	228	221
miR-745-5p	CGGCTCATCGTATGGCAGTTTGCT	19	23	23	25
miR-745-3p	CAGCTGCCTAGCGAAGGGCAACT	32	22	18	15
miR-745	CAGCTGCCTAGCGAAGGGCAACA	137	112	77	127
miR-71-5p	TGAAAGACATGGGTAGTGAGAT	58	65	49	69
miR-71-3p	TCTCACTACCTTGTCTTTCAT	11	7	3	7
miR-71	TGAAAGACATGGGTAGTGAGATG	56	42	41	66

miR-6497-5p	TCTGAGGACCGGGGCGTGT	27407	9782	21997	29811
miR-6497-3p	GGAGGCGGCCGGTGCCGGGC	8948	9981	3845	3460
miR-6496-5p	ATAGCCCAGCACTGAATCCCGCGGT	3482	2429	1918	1396
miR-6495-5p	GCCTGAGCGTTCGAGTTCCA	68	38	107	71
miR-6495-3p	TCGGAACGCGAAGAGCACC	2041	598	1854	1713
miR-6307-3p	CAAGCTCGAACTTTTCCTGCGT	21	28	28	21
miR-6098	GTAGTGAATTGTGTGTCGGATA	3	16	25	9
miR-6094	TATTCGAGACCTCTGCTGATC	203	248	312	364
miR-6058	CGACAATGGTGATGACAG	201	30	46	125
miR-6056	AAGGAACGAGGATGGGAGTT	1	63	20	10
miR-6040	TATCGGGCAGTAGTGGGAT	2480	1629	1957	1795
miR-6006-5p	TAACTGGGATAGAGGACG	44	6	1	1
miR-4981-3p	TTGTGCTCGGTAGAGCAGCGTCGTG	1334	666	5369	4875
miR-4943-5p	TTCTATTTTGTTGGTTTTCA	633	341	579	579
miR-3900-3p	AGGAGGTGAAGATGCTTTA	6	74	90	67
miR-3885-5p	GGGCGCGGCGGCGGCGCG	50	4	11	2
miR-3878-3p	ATTGTTTTGTTGTCGTGTTG	5	21	2	2
miR-3819-5p	AATATTCTATTAATTTCCA	38	34	39	24
miR-375-3p	TTTGTTCGCCCCGGCTCGTGTCG	12	10	4	7
miR-34-5p	TGGCAGTGTGGTTAGCTGGTTGTTTGTTT	817	513	882	2010
miR-34-3p	AGCCACTAACGACACTGCTCCT	1	5	2	3
miR-3389-3p	CGCCTGGGAACACCGCGTG	11379	6756	7500	8387
miR-3387-5p	AATTATTTAAAACGATCGTGT	1	9	3	1
miR-33-5p	GTGCATTGTAGTTGCATTGCA	74	30	42	53
miR-3344	TATTGCAAGAAGGACTCAGCCAGCGG	12	35	12	12

miR-3343	TCACACACTCGTTCGTGACCGTAGC	535	1053	1000	510
miR-33	GTGCATTGTAGTTGCATTG	56	138	44	54
miR-3262	AGGGCTCTGGAATAGTTGAAGA	2	4253	11312	10562
miR-31a-5p	AGGCAAGAAGTCGGCATAGCTGTA	372	327	282	536
miR-31a	GGCAAGAAGTCGGCATAGCTGTA	359	21	7	528
miR-317-5p	CGGGTGCCACGCTGTGCTCTCT	1	2	1	3
miR-317-3p	GTGAACACAGCTGGTGGTAT	251	170	246	465
miR-317	TGAACACAGCTGGTGGTATCTCA	255	193	246	456
miR-31-5p	AGGCAAGAAGTCGGCATAGCTGT	487	518	400	707
miR-308-3p	AATCACAGGATAATACTGCGAG	320	297	231	322
miR-307a-3p	TCACAACCTCCTTGAGTGA	494	97	183	256
miR-307-5p	ACTCACTCAACCTGGGTGTGATG	1	1	3	2
miR-307-3p	TCACAACCTCCTTGAGTGAGCGA	494	116	181	290
miR-307	CACAACCTCCTTGAGTGAGCGA	491	97	189	290
miR-306a-5p	CCAGGTACTAGGTGACTCTGA	1160	1305	689	1172
miR-306a-3p	AGAGCCGCCTCGTGCCTCCGCA	16	15	7	9
miR-305-5p	ATTGTACTTCATCAGGTGCTCTGG	231	157	186	211
miR-305-3p	AGGCGCTTGTTGGAGTACACTTA	370	269	337	428
miR-2c	TATCACAGCCAGCTTTGTTGACT	24	14	22	20
miR-2b-3p	TATCACAGCCAGCTTTGTT	24	10	21	23
miR-2b	TCACAGCCAGCTTTGATGAG	155	120	91	136
miR-2a-3p	TATCACAGCCAGCTTTGATGA	45	44	42	48
miR-2a-2-5p	TCACAAAGTGGTTGTGGTACG	9	4	6	3
miR-2a	TCACAGCCAGCTTTGATGAGCAT	155	150	89	134
miR-2999	CGAGTACGGACAAGACGCGCA	4	9	20	5

miR-285	TAGCACCATTCGAATTCAGTGC	16	16	8	14
miR-283-5p	AAATATCAGCTGGTAATTCTG	20	10	21	21
miR-283	AAATATCAGCTGGTAATTCTGGG	21	14	21	21
miR-282-5p	TAGCCTCTCCTTGGCTTTGTCTG	91	53	62	49
miR-2825	CGGATACGAAGAGGCTAAT	16	42	15	8
miR-282-3p	ACATAGCCTGATAGAGGTTACG	11	11	11	7
miR-282	TAGCCTCTCCTTGGCTTTGTCT	96	61	64	52
miR-2816	CTCAGTGAGGATGGAGCGT	16877	6472	8223	8138
miR-281-5p	AAGAGAGCTATCCGTCGAC	1758	1781	1335	2297
miR-281-2-5p	AAGAGAGCTATCCGTCGACAGTA	1698	1803	1263	2233
miR-281	CTGTCATGGAGTTGCTCTCTTT	2	1	4	1
miR-279d-3p	TGACTAGATTTTCACTTATCCT	32	42	40	50
miR-279d	TGACTAGATCCATACTCGTCTGC	1453	806	1370	2026
miR-279c-3p	TGACTAGATCCATACTCGTCTG	1465	1134	1434	2100
miR-279b-3p	TGACTAGATCTACACTCATTGA	1002	768	824	1221
miR-279b	TGACTAGATCTACACTCAT	1019	623	13	1244
miR-279a	TGACTAGATCCACACTCATCC	384	367	264	377
miR-2798	TGTGGCGCAGAGGATAGCGCTTTG	366	41	762	587
miR-2796-5p	AGGGGTTTCTTTCGGCCTTCA	3	2	2	5
miR-2796-3p	GTAGGCCGGCGGAAACTACTTGC	177	125	120	251
miR-2796	GTAGGCCGGCGGAAACTACT	181	83	125	261
miR-2795	CAAGTTTGGTGATACGCGGGCAC	35	25	23	22
miR-278-5p	CCGGACGAACTTCCCAGTTCGGCC	23	20	13	20
miR-278-3p	TCGGTGGGATCTTCGTCCGTTT	786	783	521	1022
miR-2780d	AAAGGTGTTAAGAAGAAGATTGTAG	15	3	10	10

miR-2779	CATCCGGCTCGAAGGACCA	2132	1980	1976	1287
miR-277-3p	TAAATGCACTATCTGGTACGACA	30	33	32	38
miR-2768-3p	TTGGTTAAGATATTGCATCGTC	20	5	14	9
miR-2767	TAAGTAAATCTCGTGCGGCTTG	2254	2213	1072	1827
miR-2766-3p	TCAGTCTTGTCGAATGGTG	10961	11683	7626	10628
miR-2766	TCAGTCTTGTCGAATGGTTT	4345	3707	3558	4620
miR-276-5p	AGCGAGGTATAGAGTTCCTACG	190	141	176	286
miR-2765	TGGTAACTCCACCACCGTTGGC	70	32	37	63
miR-2763	ATATTATGCTCATTACTTTGGA	5	8	5	6
miR-2756	TCCCTGTAGCTGCTTAGGGGCG	754	899	455	755
miR-2755-3p	CACCCTGTCAGACCATACTTGTT	1573	1836	916	2155
miR-275-3p	TCAGGTACCTGAAGTAGCGCGCG	15368	8790	7024	14004
miR-2753	CGAGTGGAAGAAGCTGGA	1229	1022	1005	1250
miR-275	TCAGGTACCTGAAGTAGCGCG	15604	6943	7301	14375
miR-274-5p	TTTGTGACCGTCACTAACGGGCAT	12	22	15	23
miR-2745	TAAATTCGTGTCATCGGAC	1542	1626	1937	1903
miR-2738	AAGATTCTTAGAACGGCCATCTGATGT	52	61	49	75
miR-2723	CAGCAGTAGACGTTCTGTGG	14	215	277	208
miR-263b-5p	CTTGGCACTGGGAGAATTCA	8	6	1	4
miR-263a-5p	AATGGCACTGGAAGAATTCACGG	5082	4468	3500	4138
miR-2526-3p	GCCTGTAGACGGTTGCGGTG	16	6	173	71
miR-252-5p	CTAAGTACTAGTGCCGCAGGAG	132	75	74	74
miR-210	CGGTGGTAGTGACAACGG	1592	4344	1501	2067
miR-190-5p	AGATATGTTTGATATTCTTGGTTG	227	216	173	241
miR-190-3p	CCCAGGAATCAAACATATTACACA	25	18	16	19

miR-190	AGATATGTTTGATATTCTTGGTTGT	223	145	164	237
miR-184b	TGGACGGAGAACTGATAAGGA	556	341	578	753
miR-184-3p	TGGACGGAGAACTGATAAGGGC	11350	8694	7966	10309
miR-184	TGGACGGAGAACTGATAAGGG	11731	7391	8450	10998
miR-1-5p	CCGTGCTTCCTTACTTCCCATA	1	8	4	4
miR-14-3p	TCAGTCTTTTTCTCTCTCCTAT	333	232	95	153
miR-14	TCAGTCTTTTTCTCTCTCCTA	309	174	89	138
miR-1-3p	TGGAATGTAAAGAAGTATGGAG	132125	70521	67556	160084
miR-13b-3p	TATCACAGCCATTTTTGACGAGT	11	6	12	8
miR-13a-5p	CTGTCAAAGCGGCGGTGAAATG	15	15	14	21
miR-13a-3p	TATCACAGCCACTTTGATGTGG	3	6	4	1
miR-133-3p	TTGGTCCCCTTCAACCAGCTGT	15	16	10	25
miR-124	TAAGGCACGCGGTGAATGCCA	14	20	12	17
miR-12	TGAGTATTACTTCAGGTACTGGT	144	102	94	106
miR-1175-5p	AAGTGGAGGTGTGATCTCTTT	104	73	124	166
miR-1175-3p	GAGATTCAACTCCTCCAACTTAA	23	19	41	24
miR-11-5p	AGAACTCCGGCTTACTCGAACTGTG	11150	4663	17199	16649
miR-11-3p	CATCACAGTCAGAGTTCTAGCT	115	105	81	98
miR-10-5p	TACCCTGTAGATCCGAATTTGT	1524	949	1162	1825
miR-10-3p	CAAATTCGGTTCTAGAGAGGTTT	2213	2447	1520	2744
miR-100	AACCCGTAGATCCGAACTTGTG	344	271	273	336
miR-10	ACCCTGTAGATCCGAATTTGT	1511	711	1155	1812
let-7-5p	TGAGGTAGTAGGTTGTATAG	8816	6480	6467	11176
let-7	TGAGGTAGTAGGTTGTATAGTA	8453	3951	5934	10162
bantam	TGAGATCATTGTGAAAGCTGA	387	311	362	456

Table S3 The potential novel miRNAs in the four libraries of Asain corn borer

microRNA ID	Precursor Sequence
ACB-AbR-1-m0001	GAGGTGTAGCATAAGTGGGAGATCGTTTCGGCGGTCGTCGCTGAAAAACCACTACTTTCATTGTTTCATTACTTACTCG
ACB-AbR-1-m0002	GAGGTGTAGCATAAGTGGGAGATCGTTTCGGCGGTCGTCGCTGAAAAACCACTACTTTCATTGTTTCATTACTTACTCG
ACB-AbR-1-m0003	AGACAGCAGTGGCATTGACTGCGACGACTCCTTCGACAAAAAATCAGACAACTTGGAAGAAGCGATCGCGGTGGATGTACAGAATGAAA
ACB-AbR-1-m0004	CGTAAGACGGAGATCCGGCTCGAAGGACCAACCGAGAACTGCCATCGGCGCCGGAAGCACTCTGCACCATTCAGGCGG
ACB-AbR-1-m0005	TAAGAAATCACGGCGCGATTTTCACCCGCGCCCGCGGCGGTTGTGGAATTGCGGTTTTATTTCAAA
ACB-AbR-1-m0006	TCTTAGCCGCGAATTTGTGCCCAACTGCCTCCAGTCAAAGACCAAATGGAACATGAGCACTGGGGAAGTGGCTTTTAGC
ACB-AbR-1-m0007	GAGGTGTAGCATAAGTGGGAGATCGTTTCGGCGGTCGTCGCTGAAAAACCACTACTTTCATTGTTTCATTACTTACTCG
ACB-AbR-1-m0008	TCCTGCTTGGTGGCGTGCGCGTTCGCCGCGTCTGTGGAAGACCAGAACGCCGAGACCCTTCGCAGCAACTCCGACAGCAGCCCCGATGGC
ACB-AbR-1-m0009	TTAGCTTTTCCGATGTTGGAAGATGGGATTTGCCGCTAAACGGGGGAAATTGCGGCTTTCAATTGGCCAAATCTC
ACB-AbR-1-m0010	GAAACTGAGTACTTCGAACAGCAGCGAGACATGGTATTCCAGCACGACAGCGCTCCCGTACATTTATCTCGTAGTTTACGAGCGCATTTGGACAT
ACB-AbR-1-m0011	GAGGTGTAGCATAAGTGGGAGATCGTTTCGGCGGTCGTCGCTGAAAAACCACTACTTTCATTGTTTCATTACTTACTCG
ACB-AbR-1-m0012	TGAGTTTAGCAATTCTGCGGACTACATCGGTCATTTCTGATTCGATCACTCAGGGAAACTCCGAGCAGTGCTCGCGCACGGATCTGAGACGTG
ACB-AbR-1-m0013	AGCTTCGGACAAATTAATTGAAGGCACAGCTTGAAATTTTAACCGCCTTTTAGTAGAATATTCACTGTGCAAATAATTGTCGGGCGAGAAGTG
ACB-AbR-1-m0014	ACAAGTTTGGAGGTCTTGACATATTAGTTTCGAACGCTGCCGTCAATCCAACCGTCGATGCCACACTAGAGACTAGTGAAGAAGCTTGGGACAAAATATTC
ACB-AbR-1-m0015	TGAGTTTAGCAATTCTGCGGACTACATCGGTCATTTCTGATTCGATCACTCAGGGAAACTCCGAGCAGTGCTCGCGCACGGATCTGAGACGTG
ACB-AbR-1-m0016	TACCCGAACAGTGAAGGGGTCGGACTGGCCGACTTGTGATCCGAGGAATGCGGGTTCGGTCCCCGCCGCCGCTTGACTATTGTGGTGA
ACB-AbR-1-m0017	GGTAGCTTGGGGTCATTGGACAGAGTTCTACGTGCTCGGCGACCAGACTTTATAAACAGGTACCACTTGCTACCA
ACB-AbR-1-m0018	ACTGCGGAGAGCGACGCATTCACACAAACGTGGCTTGTGGGTCTGCCGCTGGCTATTTGTGGTCTGCTACATTCTAACCACCCT
ACB-AbR-2-m0001	CGTAAGACGGAGATCCGGCTCGAAGGACCAACCGAGAACTGCCATCGGCGCCGGAAGCACTCTGCACCATTCAGGCGG
ACB-AbR-2-m0002	CCACCACCATTGAGCGGTTCCCCTACGCTGTGCAGGTTCAAAGAAGCAATCAGCTGACGTGTGGCGGGACGCTGCTCACAAACA
ACB-AbR-2-m0003	CCACCACCATTGAGCGGTTCCCCTACGCTGTGCAGGTTCAAAAAAGCAATCAGCTGACGTGTGGCGGGACGCTGCTCACAAACC
ACB-AbR-2-m0004	TATTACTGTAACAGTACAGCTTTAGTACACAATTCGATGCCGTGTGGGTCGGCTGTGTACTGAATAGCAGCAATAGTGTCC
ACB-AbR-2-m0005	TATTACTGTAACAGTACAGCTTTAGTACACAATTCGATGCCGTGTGGGTCGGCTGTGTACTGAATAGCAGCAATAGTGTCC

ACB-AbR-2-m0006	TTCCATGGGCATTGAACACTGAGAAGACATTTCCTTACAAAATGCCATTTTGAAAGTGCTCAAAGCTCATGTAA
ACB-AbR-2-m0007	CTCGGGTGGGTATAAAAAGTTACGAGCTATGCGAATCCAAGTCGGGATATTTGTGGAAGATGAAACTTTATGCCGGCAAAGG
ACB-AbR-2-m0008	TCTTAGCCGCGAATTTGTGCCCAACTGCCTCCAGTCAAAGACCAAATGGAACATGAGCACTGGGGAAGTGGCTTTTAGC
ACB-AbR-2-m0009	TTCCATGGGCATTGAACACTGAGAAGACATTTCCTTACAAAATGCCATTTTGAAAGTGCTCAAAGCTCATGTAA
ACB-AbR-2-m0010	TTTCATGGGCATTGAACACTGAGAAGACATTTCCTTACAAAATGCCATTTTGAAAGTGCTCAAAGCTCGTGTAA
ACB-AbR-2-m0011	CGCGCGTCGCCATCGACGCACGCGGCTACAGTCACCGCGACATGACACTGTGACGTGATAATATGAGTAGAGCCGAAGTGTCGGCGGAAGCC
ACB-AbR-2-m0012	TTTTTGGCACTAGGAACATTGTGGATAGAAAGCTGGGAGAACTTTGGGCAGTTTCTAAAACCCCTTGCTTTTTCATTTGTGATATGGCCGTGGTCATATC
ACB-AbR-2-m0013	GAAACTGAGTACTTCGAACAGCAGCGAGACATGGTATTCCAGCACGACAGCGCTCCCGTACATTTATCTCGTAGTTTACGAGCGCATTTGGACAT
ACB-AbR-2-m0014	TGAATCTGTCCGATGAAAGTGCGGTTCACTTCACTTCCTGTACGCTATCCTGGCGAGGTGTCTGGAGTGCAGCGTCATACAGATTT
ACB-AbR-2-m0015	CACGTGGCGGTGCTCGACTTCTCGAAGGCATTCGACACAGTGTCCCACAAGGCACTTGTGGAGGTGCTTAGAGGGCGGGGGCTGCCCGGG
ACB-AbR-2-m0016	TGAATCTGTCCGATGAAAGTGCGGTTCACTTCACTTCCTGTACGCTATCCTGGCGAGGTGTCTGGAGTGCAGCGTCATACAGATTT
ACB-AbR-2-m0017	CACGTGGCGGTGCTCGACTTCTCGAAGGCATTCGACACAGTGTCCCACAAGGCACTTGTGGAGGTGCTTAGAGGGCGGGGGCTGCCCGGG
ACB-AbR-2-m0018	GTCGTCCATTTGCGACGACAACGTGACATCTTCTGTGTTAATCTAATTGACGATGTCACACTGTCGTCGCAAATGGACGAT
ACB-AbR-2-m0019	TACCCGAACAGTGAAGGGGTCGGACTGGCCGACTTGTGATCCGAGGAATGCGGGTTCGGTCCCCGCCGCCGCTTGACTATTGT
ACB-AbR-2-m0020	CTCGGGTGGGTATAAAAAGTTACGAGCTATGCGAATCCAAGTCGGGATATTTGTGGAAGATGAAACTTTATGCCGGCAAAGG
ACB-AbR-2-m0021	GGTAGCTTGGGGTCATTGGACAGAGTTCTACGTGCTCGGCGACCAGACTTTATAAACAGGTACCACTTGCTACCA
ACB-AbR-2-m0022	ACTGCGGAGAGCGACGCATTCACACAAACGTGGCTTGTGGGTCTGCCGCTGGCTATTTGTGGTCTGCTACATTCTAACCACCCT
ACB-BtS-1-m0001	AGACGTAGCGTGGGCAGGCCTCCATTTGCCTCTGGTAAATTAGAGGGTCGTGCTCCTGCAGTGGAG
ACB-BtS-1-m0002	GGCGCGCGCGCTTGTGATTTGTTGAGCAGTTAATACCAACTGCTCGAGCAGTTATCGCTGGCAGCTTCAAGCTG
ACB-BtS-1-m0003	GGCGCGCGCGCTTGTGATTTGTTGAGCAGTTAATACCGACTGCTCGAGCAGTTATCGCTGGCAGCTTCAAGCTG
ACB-BtS-1-m0004	GGCGCGCGCGCTTGTGATTTGTTGAGCAGTTAATACCAACTGCTCGAGCAGTTATCGCTGGCAGCTTCAAGCTG
ACB-BtS-1-m0005	GGCGCGCGCGCTTGTGATTTGTTGAGCAGTTAATACCGACTGCTCGAGCAGTTATCGCTGGCAGCTTCAAGCTG
ACB-BtS-1-m0006	ACAACCAATCTGTATGAACTGCTAGGGAGCGAGCGATTCCTGCGGACCGCATTACTCTAAAACGCTTCCTCGAAGTTTACAGATCGGCTGTG
ACB-BtS-1-m0007	GAGGTGTAGCATAAGTGGGAGATCGTTTCGGCGGTCGTCGCTGAAAAACCACTACTTTCATTGTTTCATTACTTACTCG
ACB-BtS-1-m0008	GAGGTGTAGCATAAGTGGGAGATCGTTTCGGCGGTCGTCGCTGAAAAACCACTACTTTCATTGTTTCATTACTTACTCG

ACB-BtS-1-m0009	ATATCATCAGAGACGCTATCACCGTCATCGACCGCAACAGCATCGGTGATGAATGGGTAGTTTTCAACAAACAA
ACB-BtS-1-m0010	TGTACTACCAGTTCGATGAATACGGCTGGTGGTGGTTCTTCCTGCAATTCCCGGTTGTCTTTATTTATTCTGACTACAC
ACB-BtS-1-m0011	TGTACTACCAGTTCGATGAATACGGCTGGTGGTGGTTCTTCCTGCAATTCCCGGTTGTCTTTATTTATTCTGACTACAC
ACB-BtS-1-m0012	CGTAAGACGGAGATCCGGCTCGAAGGACCAACCGAGAACTGCCATCGGCGCCGGAAGCACTCTGCACCATTCAGGCGG
ACB-BtS-1-m0013	ACATTGACGGCGCAAACCTTCCTCTGATGTTTGCCCCCGAAAGTGGTAACATGTTGGGAGGCACTGTGGTGAACATCA
ACB-BtS-1-m0014	ATCGTGCTCTTGCGACGACTGTGACATCTACGTTAATCTAATTGGAGATGTCACACTGTCGTCGCAAATGGACGAA
ACB-BtS-1-m0015	TCTTAGCCGCGAATTTGTGCCCAACTGCCTCCAGTCAAAGACCAAATGGAACATGAGCACTGGGGAAGTGGCTTTTAGC
ACB-BtS-1-m0016	GAGGTGTAGCATAAGTGGGAGATCGTTTCGGCGGTCGTCGCTGAAAAACCACTACTTTCATTGTTTCATTACTTACTCG
ACB-BtS-1-m0017	GGCTCGAGCGCGCCGGCGGCGACCTGGACGCGGCGTACAAGTTCCTAGACGCCGCCGGCTCCAAGCTGGACTACCG
ACB-BtS-1-m0018	CATCCAGATCAGCATCAGGACTGGGATGGTCGCTTTTTCTGTTGCCATAGGAGCTGCATTCCCGAGCTTAGAGCTGGTCATCAGCTTCGTTGGAGC
ACB-BtS-1-m0019	GGCTCGAGCGCGCCGGCGGCGACCTGGACGCGGCGTACAAGTTCCTAGACGCCGCCGGCTCCAAGCTGGACTACCG
ACB-BtS-1-m0020	AATCGTGCTCTTGCGACGACTGTGACATCTACGTTAATCTAATTGGAGATGTCACACTGTCGTCGCAAATGGACGAAA
ACB-BtS-1-m0021	GAAACTGAGTACTTCGAACAGCAGCGAGACATGGTATTCCAGCACGACAGCGCTCCCGTACATTTATCTCGTAGTTTACGAGCGCATTTGGACAT
ACB-BtS-1-m0022	GAGGTGTAGCATAAGTGGGAGATCGTTTCGGCGGTCGTCGCTGAAAAACCACTACTTTCATTGTTTCATTACTTACTCG
ACB-BtS-1-m0023	TACCCGAACAGTGAAGGGGTCGGACTGGCCGACTTGTGATCCGAGGAATGCGGGTTCGGTCCCCGCCGCCGCTTGACTA
ACB-BtS-1-m0024	AGCACGATGATATTGTCGTAGACGCGAGAATTTATAGAATTCTCGATGTAGGGACGCTCGCTGGCACGTGAC
ACB-BtS-1-m0025	CCACAAAAAATGAGCATCCCGGGTATAGAGACTTTAGATCACTCAAGAAACCCGGGATCTCATCAGACACCACAC
ACB-BtS-1-m0026	TGGTTACCTCTTCGGAAGTCAAGGGAGATACTTTCAAGGGTTTTCTTAAGAACCCTGGTTTGAATGTATGATTCAGTGGATACGGATAGCCCGAGA
ACB-BtS-1-m0027	AATCGTGCTCTTGCGACGACTGTGACATCTACGTTAATCTAATTGGAGATGTCACACTGTCGTCGCAAATGGACGAAA
ACB-BtS-1-m0028	AATCGTGCTCTTGCGACGACTGTGACATCTACGTTAATCTAATTGGAGATGTCACACTGTCGTCGCAAATGGACGAAA
ACB-BtS-1-m0029	AATCGTGCTCTTGCGACGACTGTGACATCTACGTTAATCTAATTGGAGATGTCACACTGTCGTCGCAAATGGACGAAA
ACB-BtS-1-m0030	GGTAGCTTGGGGTCATTGGACAGAGTTCTACGTGCTCGGCGACCAGACTTTATAAACAGGTACCACTTGCTACCA
ACB-BtS-1-m0031	GGAATATTATATCAGTGAAACGCCCGTGTTGAGGCAGTTACTCTTGACGCCATGCAACTCCGTACGTTTCTCACATTTATAAAAAAGC
ACB-BtS-1-m0032	ACAGCCAATCTGTATGAACTGCTAGGGAGCGAGCGATTCCTGCGGACCGCATTACTCTAAAACGCTTCCTCGAAGTTTACAGATCGGCTGTG
ACB-BtS-2-m0001	GAGGTGTAGCATAAGTGGGAGATCGTTTCGGCGGTCGTCGCTGAAAAACCACTACTTTCATTGTTTCATTACTTACTCG

ACB-BtS-2-m0002	GAGGTGTAGCATAAGTGGGAGATCGTTTCGGCGGTCGTCGCTGAAAAACCACTACTTTCATTGTTTCATTACTTACTCG
ACB-BtS-2-m0003	CGTAAGACGGAGATCCGGCTCGAAGGACCAACCGAGAACTGCCATCGGCGCCGGAAGCACTCTGCACCATTCAGGCGG
ACB-BtS-2-m0004	TGGTAATTCACCGTCTGGCACTAAGTTCCAAGCCAATCTGCTGGTGATCACCAACTCAGCGTGCCAGGGAACCTGGATGCCCGGCATCGTCATCGCG
ACB-BtS-2-m0005	TCTTAGCCGCGAATTTGTGCCCAACTGCCTCCAGTCAAAGACCAAATGGAACATGAGCACTGGGGAAGTGGCTTTTAGC
ACB-BtS-2-m0006	ACCCAAAGTGGCCAAAAATTTGACACAACCTTATTCCAATTGTAATAAAGACGTGTTGCGAACTTTTTGGCTGCTTTGGG
ACB-BtS-2-m0007	GAGGTGTAGCATAAGTGGGAGATCGTTTCGGCGGTCGTCGCTGAAAAACCACTACTTTCATTGTTTCATTACTTACTCG
ACB-BtS-2-m0008	GTTCTTTAGCCGTATGGGCGGAGAAAATGGTAGAAAACGCGGAGTCTCTACCATCTTGGTTGTCAGCT
ACB-BtS-2-m0009	TAAAGGTGGTTTTATATTTGTCTGATGTGTCGTGACGTGACGCGTCAGAACTGTATCGGTTCCATACATA
ACB-BtS-2-m0010	TAAAGGTGGTTTTATATTTGTCTGATGTGTCGTGACGTGACGCGTCAGAACTGTATCGGTTCCATACATA
ACB-BtS-2-m0011	GAAACTGAGTACTTCGAACAGCAGCGAGACATGGTATTCCAGCACGACAGCGCTCCCGTACATTTATCTCGTAGTTTACGAGCGCATTTGGACAT
ACB-BtS-2-m0012	GAGGTGTAGCATAAGTGGGAGATCGTTTCGGCGGTCGTCGCTGAAAAACCACTACTTTCATTGTTTCATTACTTACTCG
ACB-BtS-2-m0013	GTCGTCCATTTGCGACGACAACGTGACATCTTCTGTGTTAATCTAATTGACGATGTCACACTGTCGTCGCAAATGGACGA
ACB-BtS-2-m0014	CAGGAGTTGCAGCGGTGGGAACGGGAGGTGGGAATAATCGCATTGCAAGTTTACACATCGTTTCAGCCTAC
ACB-BtS-2-m0015	GGGCTGGAAGACGTAGCGTGGGCTGATGGTTTATTGTAGAGTGAGGTAATGCATGTCTTGCTGCCCA
ACB-BtS-2-m0016	ACGTACACCAGTTCTTTAGCTCGTCCCCAGACGTAAAAATCGAACGGAGTTAGGTCTGGGGAACGTGCAGGCCACTGTATAG

Table S4 The common and specially expressed novel miRNAs in Asian corn borer

miRNA ID	Reads	miRNA ID	Reads	miRNA ID	Reads	miRNA ID	Reads	Sequence
ACB-BtS-1-m0012_5p	43	ACB-BtS-2-m0003_5p	21	ACB-AbR-1-m0004_5p	42	ACB-AbR-2-m0001_5p	21	AGATCCGGCTCGAAGGACCA
ACB-BtS-1-m0015_3p	272	ACB-BtS-2-m0005_3p	99	ACB-AbR-1-m0006_3p	71	ACB-AbR-2-m0008_3p	142	TGGAACATGAGCACTGGGGAAGT
ACB-BtS-1-m0021_5p	37	ACB-BtS-2-m0011_5p	30	ACB-AbR-1-m0010_5p	14	ACB-AbR-2-m0013_5p	26	ACTTCGAACAGCAGCGAGACAT
ACB-BtS-1-m0007_5p	378617	ACB-BtS-2-m0001_5p	334760	ACB-AbR-1-m0001_5p	307019	non		ATAAGTGGGAGATCGTTTCGGCG
ACB-BtS-1-m0008_5p	378617	ACB-BtS-2-m0002_5p	334760	ACB-AbR-1-m0002_5p	307019	non		ATAAGTGGGAGATCGTTTCGGCG
ACB-BtS-1-m0016_5p	378617	ACB-BtS-2-m0007_5p	334760	ACB-AbR-1-m0007_5p	307019	non		ATAAGTGGGAGATCGTTTCGGCG
ACB-BtS-1-m0022_5p	378617	ACB-BtS-2-m0012_5p	334760	ACB-AbR-1-m0011_5p	307019	non		ATAAGTGGGAGATCGTTTCGGCG

ACB-BtS-1-m0030_5p	9	non		ACB-AbR-1-m0017_5p	9	ACB-AbR-2-m0021_5p	7	GTAGCTTGGGGTCATTGGACA
non		ACB-BtS-2-m0013_3p	13	non		ACB-AbR-2-m0018_3p	23	CGATGTCACACTGTCGTCGCA
non		non		ACB-AbR-1-m0018_3p	9	ACB-AbR-2-m0022_3p	6	GCTATTTGTGGTCTGCTACATTC

Table S5 The different expression of known miRNA between Cry1Ab susceptible and resistant Asian corn borer (separate Excel documents)

Table S6 The different expression of novel miRNA between Cry1Ab susceptible and resistant Asian corn borer (separate Excel documents)

Table S7 The statistic of target gene of miRNAs from the four libraries of Asian corn borer

	Sample	miRNA	Target site	Target gene
Known miRNAs	ACB-BtS-1	302	1865157	72831
	ACB-BtS-2	395	2367013	72830
	ACB-AbR-1	268	1555935	72817
	ACB-AbR-2	287	1647616	72815
Novel miRNAs	ACB-BtS-1	20	118999	54605
	ACB-BtS-2	12	67712	41616
	ACB-AbR-1	14	72875	43515
	ACB-AbR-2	15	97442	50976

Table S8 The GO terms for known different miRNAs from the component ontology with p-value as good or better than 1 (separate Excel documents)

Table S9 The GO terms for known different miRNAs from the function ontology with p-value as good or better than 1 (separate Excel documents)

Table S10 The GO terms for known different miRNAs from the process ontology with p-value as good or better than 1 (separate Excel documents)

Table S11 The GO terms for novel different miRNAs from the component ontology with p-value as good or better than 1 (separate Excel documents)

Table S12 The GO terms for novel different miRNAs from the function ontology with p-value as good or better than 1 (separate Excel documents)

Table S13 The GO terms for novel different miRNAs from the process ontology with p-value as good or better than 1 (separate Excel documents)

Table S14 The pathway annotation of known differentially expressed miRNAs (separate Excel documents)

Table S15 The pathway annotation of novel differentially expressed miRNAs (separate Excel documents)

Table S16 The pimers used in the study for quantitative real-time PCR

miRNA name	RT primer (5′-3′)	Forward primer (5′-3′)	Reverse primer (5′-3′)
miR-3477-5p	CTCAACTGGTGTCGTGGAGTCGGCAATTCAGTTGAGCTCACAGT	GGTAGGTAATCTCATTTGGTA	AACTGGTGTCGTGGAGTCGGC
miR-6017-5p	CTCAACTGGTGTCGTGGAGTCGGCAATTCAGTTGAGTAAAAATA	CTGGTAGGTTAGGATTTG	AACTGGTGTCGTGGAGTCGGC
miR-4954-3p	CTCAACTGGTGTCGTGGAGTCGGCAATTCAGTTGAGGAGTCCTC	TAGGTGATCCGGTTCGAG	AACTGGTGTCGTGGAGTCGGC
miR-315-5p	CTCAACTGGTGTCGTGGAGTCGGCAATTCAGTTGAGCTTTGTGC	GGTAGGTTTTGATTGTTG	AACTGGTGTCGTGGAGTCGGC
miR-3352	CTCAACTGGTGTCGTGGAGTCGGCAATTCAGTTGAGAACAAAAT	CTGGTAGGTGGTCTCGGTTCT	AACTGGTGTCGTGGAGTCGGC
miR-315b	CTCAACTGGTGTCGTGGAGTCGGCAATTCAGTTGAGGTGCAGTA	GGTAGGTTTTGATTGTTG	AACTGGTGTCGTGGAGTCGGC
miR-2548-5p	CTCAACTGGTGTCGTGGAGTCGGCAATTCAGTTGAGTGCCAACA	TAGGTAGGGATAACTGTTGG	AACTGGTGTCGTGGAGTCGGC
miR-3275	CTCAACTGGTGTCGTGGAGTCGGCAATTCAGTTGAGTGAAGAAC	CTGGTAGGTGATTCGTTG	AACTGGTGTCGTGGAGTCGGC
U6	AACTGGTGTCGTGGAGTCGGC	CCATACCACCCTGGAAACGC	TACTAACCGAGCCCGACCCT
